# Observations on Affections of the Mammœ, Liable to Be Mistaken for Cancer

**Published:** 1828-07

**Authors:** John Parrish

**Affiliations:** One of the Surgeons of the Pennsylvania Hospital.


### THE MAMMÆ.

*Observations on Affections of the Mammæ, liable to be mistaken for Cancer.* By John Parrish, m.d. one of the Surgeons of the Pennsylvania Hospital.

From much experience in cancerous affections of the breast, I have been led to the conclusion that derangements of structure in that organ, of a nature comparatively innocent, are often mistaken for scirrhus. Not a little of the success which has been attributed to surgical treatment in this terrible complaint has, I believe, arisen from its direction to such cases. My own opinion is, that real cancer is a constitutional affection, and that, in the greater number of instances, even extirpation affords but a slight chance of ultimate safety to the patient. On a future occasion, I may perhaps offer the results of my observation on this point: at present my object is to illustrate, by a detail of facts, the liability of the surgeon to misapprehend the character of tumors in the mamma, and thus unnecessarily to subject the female to severe pain, and to a mutilation which must always be unpleasant, and to a young person often very inconvenient.

I will begin by stating, that even the natural structure of the mamma may be mistaken for scirrhus. Every practitioner who has often been consulted in the complaints of females must have observed that in some women there is an unusual hardness of the breast, without any departure from a perfectly healthy condition. This hardness is occasionally attended with a lobular structure of the gland; and it is not uncommon for the young surgeon to confound this state of things with disease, and to suppose that he is handling a cancerous tumor, when the parts are in reality perfectly sound. Even the experienced hand is not always secure from such a mistake.

I recollect well the case of a lady of this city, who came under the care of my lamented preceptor, the late Dr. Wistar, and myself, with pain in the breast. We made repeated examination, and, though aware of the occasional hardness of the mamma, were convinced that we could feel a tumor. As the patient complained of much pain, which she referred to a particular spot, and this spot coincided with that in which we supposed the tumor to exist, we concluded to recommend an operation. The patient consented; but in the mean time her mind was so strongly impressed by a dream, in which she thought that a cure might be effected by the application of a certain nostrum, that she was unwilling to submit to the knife till the remedy had been tried. We were not very reluctant to suspend the operation; and it was agreed that she should be allowed time to satisfy her mind. Not long afterwards, Dr. Wistar was attacked by the illness of which he died; and the operation was on this account still further delayed. Sometime after his death, I was called in to see her. Upon making a strict investigation into her case, I was now convinced that the pain in her breast arose from a rheumatic affection, and that what had been taken for a tumor was nothing more than a portion of the mamma unusually firm. The idea of an operation was therefore relinquished: the pain gradually subsided under treatment calculated for the cure of rheumatism; and, though several years have elapsed, there has been no evidence afforded of the existence of cancerous disease.

In another case I was on the point of committing an error which would have caused me much uneasiness, and which I now mention in the hope that my experience may prove useful to others. In the year 1819, I was called to see a young woman with a small tumor in her breast, which so far presented the characters of scirrhus that I conceived its extirpation advisable. The patient very willingly consented. At the time appointed, I went to the house with two of my medical friends, who attended in order to witness and assist in the operation. Before proceeding to use the knife, I placed my hand upon her breast, and was surprised to find, as I thought, that the tumor had much increased in size. My friends who were present also examined it, and thought they could feel a tumor of considerable magnitude. I had even traced the course and extent of the proposed incision, and was about to commence the operation, when, almost by accident, I touched the real tumor, which remained exactly as when I first examined it. I had mistaken the natural hardness of the breast for scirrhous induration.

So much for the liability of mistaking a natural healthy condition of the breast for scirrhus. The difficulty of forming a correct judgment is much increased when tumors really exist. It by no means follows that every hard, chronic, painful tumor of the mamma is cancerous. In the young female, if the breast remain freely moveable, and the skin present the natural aspect, even though the ordinary symptoms of scirrhus be exhibited, there is much reason to hope that the complaint is of another character, and may be relieved without the necessity of an operation.

I have twice attended a lady affected with a tumor in the mamma. On both occasions it was large, very painful, and of a chronic character, and caused much alarm to herself and her friends; but, by the use of purging, low diet, and discutient applications, it was entirely dispersed.

In another instance, a young woman was placed under my care with a tumor of the breast, accompanied with enlargement of the axillary glands. In this case the discutient plan was employed, without any obvious effect. Dr. Wistar was called in consultation. It was resolved that the tumor should be removed, and the glands of the axilla dissected out. The patient was sent to the country for a short time, and, on her return, I found a very evident abatement of the tumor. This seldom, if ever, happens in genuine scirrhus, and I felt much encouraged as to the result of her case. Though the young woman herself was still desirous that something should be done, I determined for the present not to operate. Between one and two months afterwards, Dr. Wistar again met me in consultation; and it was our decided opinion that the complaint was considerably better. The young woman was about to be married, and had a very natural dread of entering into the new state with a diseased breast. The circumstance was made known to us, and we were desired to decide upon the proper course,—whether the operation should be performed, or the marriage should take place in her present condition. We gave the subject full consideration, and concluded to recommend that she should go home, and wait for a short time the result of that change which appeared to have commenced. Should the tumor increase, she might then come to the city, and have it removed. She followed our advice. The complaint gradually disappeared; she was married; and, when I last heard from her, was the healthy mother of several children. This is a case in which, had an operation been performed when first proposed, we should have honestly attributed to it the credit of the cure.

When, in young persons, instead of a single tumor, there are two or more in the breast, the probability that they are not cancerous is still greater. It is not uncommon for practitioners to be consulted in cases of this kind. Sometimes both mammæ are affected, sometimes only one.

A young woman was supposed to have a cancer in her breast. Dr. Physick and myself attended her. There were two tumors in one breast, and one in the other. We had before seen a case somewhat similar, in which the complaint had disappeared without an operation; but, in the present, it was impossible to decide with certainty whether the tumors were cancerous or not, as they were not sufficiently advanced to display the requisite characters. We resolved, however, to postpone an operation till efforts had been made to discuss them. A low diet, with the use of saline purgatives, and a mercurial plaster to the breast, were recommended; and the patient was advised to go home, and return to the city if there should be any increase of the complaint. Instead, however, of increasing, the tumors gradually diminished, and at length disappeared; nor have they since returned.

Scrofulous affections of the breast not unfrequently occur, and are sometimes confounded with cancer. Several cases of this kind have fallen under my observation.

A young married woman, many years ago, was sent to me from Bucks County, under the impression that she had a cancer of the mamma, which required an operation. Her breast was considerably enlarged, presenting an irregular surface, with redness of the skin. The axillary glands were also diseased. Considering that, if the complaint were cancerous, it had proceeded so far that an operation would be of very doubtful success, I deferred it for a short time. Happily the tumor became soft, and fluctuation was observable. It was opened, and scrofulous matter was discharged. The patient returned to the country, and gradually regained her health. In this instance there was none of that contraction or wrinkling of the skin which generally accompanies genuine cancer when it has advanced to the surface. But, as the disease is sometimes attended with a swelling of the breast and a smooth skin, this circumstance did not enable me to form a certain diagnosis. Subsequent observation, however, has taught me that there are peculiarities in the appearance of a scirrhous breast, even when swollen and smooth, by which it may generally be distinguished. Instead of a diffused redness, I have usually noticed, under these circumstances, a stellated appearance of the skin, red spots being scattered like spangles over the surface. This peculiarity is so striking, that I believe I could now distinguish, by the eye alone, between such tumors and mere scrofulous enlargement. The experience acquired from this case was of great use to me in one which afterwards occurred.

Sometimes deep-seated abscesses, of a scrofulous nature, form in the breast, and, being attended with much pain, are liable, without care, to be mistaken for scirrhus. Two very interesting cases of this kind have occurred to me, both in young married women, with infant children.

The subject of the first case was sent to me from the country. She had been for several months affected with pain in her breast. Some treatment had been employed for her relief, but without success, and a tumor had formed. When I first saw her, I examined the breast carefully, and I thought I could feel a deep-seated, indistinct fluctuation. This circumstance was sufficient to deter me from operating, till I had satisfactorily ascertained the nature of the tumor. I stated to her friends my hopes that the complaint might prove to be scrofulous, and proposed that a lancet should be introduced into the tumor, in order to ascertain the fact. Even should I be disappointed in my opinion, little additional pain would be occasioned, and the operation might then proceed as well as though nothing had been previously done. Their consent having been readily given, I took the lancet, and, in order that I might be certain to reach the affected part, plunged it up to the hilt in the breast, in the direction of the tumor. When I withdrew it, only a drop or two of blood followed, and I began to fear that my hopes had been unfounded; but the blood soon began to flow more freely, and a little pus made its appearance at the orifice of the wound. I enlarged the opening, and was delighted to perceive that scrofulous matter escaped freely.* The parts healed; and the complaint ultimately disappeared, leaving the breast perfectly sound.

The second case was equally interesting. I suspected that the complaint might be a deep-seated scrofulous abscess, for I thought I could feel fluctuation; and yet any surgeon, not on his guard, might have been led into error, as the fluctuation was very indistinct. I obtained permission, as in the former case, to explore the tumor before attempting an operation. Scrofulous matter immediately followed the withdrawal

* [Genuine pus, so far as Dr. Parrish has observed, is never found in cancerous affections. —EDITOR.]

of the lancet. The breast healed, and occasioned no further trouble to the patient.

In all deep-seated tumors of the breast, where the evidences of cancer are not unequivocal, I would advise the adoption of the plan pursued in the instances above mentioned. An experienced surgeon, with a delicate sense of touch, can generally detect fluctuation, if there be a collection of matter: but should he be unable to do so, what is more safe and easy than to make a puncture with his lancet before commencing the operation. If an abscess exist, it is thus rendered evident; if not, and extirpation becomes necessary, no harm has been done. I believe that such cases have often been mistaken for cancer, and operations performed without the least necessity.

Cysts occasionally form in the breast which are totally free from any cancerous disposition. Within the last year, a middle-aged woman from the country came under my care, with tumor of the mamma. Upon a careful examination, I discovered an obscure, deep-seated fluctuation. There were two tumors. I punctured them both with the lancet, and a serous fluid escaped. No unpleasant consequences resulted. Such an affection might have been readily confounded with scirrhus, and the mamma have been extirpated.

I will close the paper by observing, that we are liable to be deceived as to the nature of the action which sometimes takes place in the cicatrix, formed after the extraction of a cancerous tumor. If it be observed to enlarge, thicken, and assume a red colour, at the same time becoming tender and painful, there is reason to fear that the complaint has returned. But it occasionally becomes very painful, without undergoing any change in appearance or structure. In such cases, the disorder is probably purely nervous, and a resort to the knife wholly unnecessary. I once witnessed a case of this kind under the care of another surgeon. The pain was so severe and continued, that it was determined to remove the cicatrix, under the impression that the parts beneath might be found in a diseased state. But they were perfectly healthy, and the pain, in all probability, arose from nervous irritation.*

* Condensed from the North American Medical and Surgical Journal, for April 1828.

